# Overexpression of PELP1 in Lung Adenocarcinoma Promoted E_2_ Induced Proliferation, Migration and Invasion of the Tumor Cells and Predicted a Worse Outcome of the Patients

**DOI:** 10.3389/pore.2021.582443

**Published:** 2021-04-02

**Authors:** Dongmei Zhang, Jiali Dai, Yu Pan, Xiuli Wang, Juanjuan Qiao, Hironobu Sasano, Baoshan Zhao, Keely M. McNamara, Xue Guan, Lili Liu, Yanzhi Zhang, Monica S. M. Chan, Shuwen Cao, Ming Liu, Sihang Song, Lin Wang

**Affiliations:** ^1^ Department of Pathology, Harbin Medical University-Daqing, Daqing, China; ^2^ Traditional Psychological Unit, The Third Hospital of Daqing, Daqing, China; ^3^ Department of Pathology, Tohoku University School of Medicine, Sendai, Japan; ^4^ Department of Pathology, Daqing Oilfield General Hospital, Daqing, China; ^5^ Department of Pathology, The Fifth Affiliated Hospital of Harbin Medical University, Daqing, China

**Keywords:** estrogen, proline-, glutamic acid-, leucine-rich protein 1, lung adenocarcinoma, tumor progression

## Abstract

The expression of Proline-, glutamic acid-, and leucine-rich protein 1 (PELP1) has been reported to be dysregulated in non-small cell lung carcinoma, especially in lung adenocarcinoma (LUAD). Therefore, we aimed to investigate the functional and prognostic roles of PELP1 in LUAD in this study. We first immunolocalized PELP1 in 76 cases of LUAD and 17 non-pathological or tumorous lung (NTL) tissue specimens and correlated the findings with the clinicopathological parameters of the patients. We then performed *in vitro* analysis including MTT, flow cytometry, wound healing, and transwell assays in order to further explore the biological roles of PELP1 in 17-β-estradiol (E_2_) induced cell proliferation, migration, and invasion of LUAD cells. We subsequently evaluated the prognostic significance of PELP1 in LUAD patients using the online survival analysis tool Kaplan-Meier Plotter. The status of PELP1 immunoreactivity in LUAD was significantly higher than that in the NTL tissues and significantly positively correlated with less differentiated features of carcinoma cells, positive lymph node metastasis, higher clinical stage as well as the status of ERα, ERβ, and PCNA. *In vitro* study did reveal that E_2_ promoted cell proliferation and migration and elevated PELP1 protein level in PELP1-high A549 and H1975 cells but not in PELP1-low H-1299 cells. Knock down of PELP1 significantly attenuated E_2_ induced cell proliferation, colony formation, cell cycle progress as well as migration and invasion of A549 and H1975 cells. Kaplan-Meier Plotter revealed that LUAD cases harboring higher PELP1 expression had significantly shorter overall survival. In summary, PELP1 played a pivotal role in the estrogen-induced aggressive transformation of LUAD and could represent adverse clinical outcome of the LUAD patients.

## Introduction

Lung cancer is the leading cause of cancer mortality worldwide [1]. Non-small cell lung carcinoma (NSCLC) accounts for approximately 80% of all pathological subtypes of lung cancer and most NSCLCs are either adenocarcinomas (LUAD) or squamous cell carcinomas (LUSC). It is well known that the proliferation and metastasis of NSCLC cells, especially LUAD cells, are stimulated by estrogen *in vitro* [2–6]. In addition, ERα, ERβ and aromatase (a key enzyme for estrogen biosynthesis) status in LUAD patients was also reported to be associated with adverse clinical outcome of the patients [7–9]. These findings did indicate a critical role of estrogen signaling in the development and progression of some LUAD patients.

The biological actions of estrogen *via* ERs have been well known to require the assistance of co-regulatory proteins, including their co-activators and co-suppressors, which usually form complexes with activated ERs and determine the magnitude or specificity of ER signaling. Accumulating evidence suggests, however, that dysregulation of ER co-regulators could contribute to cancer initiation, progression, and metastasis even compared to abnormalities of ERs themselves [10–13]. For example, amplified in breast cancer 1 (AIB1), a well-documented ER co-activator, has been reported to play an important role in proliferation and invasion of lung carcinoma cells and to represent a possible prognostic marker in NSCLC patients [14, 15].

Proline-, glutamic acid- and leucine-rich protein 1 (PELP1), also known as the modulator of non-genomic actions of estrogen receptor (MNAR), is a novel ER co-regulator with distinctive characteristics from other ER coregulators. The protein structure of PELP1 contains several motifs and domains involved in the interaction with steroidal receptors, including ERs, androgen receptors, glucocorticoid receptors, and progesterone receptors. In ER related signaling pathway, PELP1 has been demonstrated to serve as a scaffold protein which could modulate various signaling complexes with ERs and participate in both genomic and non-genomic functions of them [16, 17]. It is widely accepted that PELP1 acts as a proto-oncogene. Therefore, PELP1 overexpression was reported to induce malignant transformation of normal cells, accelerate cell cycle progression, promote tumor cell proliferation, and enhance migration and invasion of tumor cells [18–20]. PELP1 expression was also reported to be dysregulated in several human malignancies, including breast [21, 22], endometrial [23], ovarian [24], colorectal [25], gastric [26], and salivary duct carcinoma [27]. In lung cancer, Marquez-Garban et al. reported overexpression of PELP1 in cultured NSCLC cell lines [28]. Slowikowski et al. also reported increased PELP1 mRNA and protein levels which were detected by real-time quantitative PCR (RT-qPCR) and western blotting in NSCLC tissues, respectively [29] and Ohshiro et al. demonstrated that PELP1 was involved in phytoestrogen resveratrol induced autophagy of lung carcinoma cells [30]. These results above all indicated that PELP1 expression was dysregulated and could play a role in the progression of NSCLC, especially in the cases of LUAD. However, the correlation between PELP1 and ERs status in LUAD, the possible biological function of PELP1 in estradiol (E_2_) regulated proliferation, migration, and invasion of LUAD cells, as well as the prognostic meaning of PELP1 in LUAD have all remained virtually unknown.

Therefore, in this study, we first assessed the PELP1 protein expression profiles in 76 primary LUAD tissues using immunohistochemistry (IHC) and correlated PELP1 with clinicopathological variables of the patients, including the status of ERs. We then explored the roles of PELP1 in E_2_ stimulated proliferation, migration, and invasion of LUAD cells. We subsequently explored the prognostic impact of PELP1 in LUAD using the online survival analysis tool Kaplan-Meier Plotter.

## Materials and Methods

### Patient Samples

A total of 76 cases of LUAD were examined in our present study, which consisted of 16 cases from the Fifth Affiliated Hospital of Harbin Medical University from 2009 to 2014; 18 from Daqing Oilfield General Hospital from 2008 to 2017; 42 from the Daqing Longnan Hospital from 2018 to 2019. Clinicopathological findings including their gender, age, smoking history, histological type, tumor size, tumor differentiation, lymph node status, and clinical stage were all retrieved from the medical records at these three institutions. All the specimens examined were retrieved from surgical pathology files of resected specimens, and all the patients were treatment naïve before the surgery. Hematoxylin and eosin stained tissue slides were all independently evaluated by two of the authors (BZ and LL) blinded to the clinical data of the patients. In addition, 17 cases of non-pathological or non-tumorous lung (NTL) tissues obtained by autopsy were retrieved from the Forensic Center of Harbin Medical University-Daqing as controls.

The research protocol of our present study was approved by the Institutional Review Board of Harbin Medical University-Daqing (Approval number: HMUDQ2020120501). All procedures were conducted in Accordance with the Declaration of Helsinki and International Ethical Guidelines for Biomedical Research Involving Human Subjects (CIOMS).

### Cells and Reagents

The human LUAD cell lines (A549, H1975, and H-1299) and the breast carcinoma cell lines (MCF-7 and MD-MBA-231) used in this study were purchased from the Cell Bank of the Chinese Academy of Sciences. Cell line authenticity was verified using STR analysis and mycoplasma was tested by the supplier prior to their shipment. The cells were maintained in DMEM medium (Gibco, Cat# 11995065) supplemented with 10% heat-inactivated fetal bovine serum (FBS, Biological Industries) at 37°C with 5% CO_2_. Before E_2_ treatment, the medium was changed to complete phenol-red free DMEM (Gibco, Cat# 21063029) supplemented with 5% charcoal stripped FBS (Hyclone, Cat# SH30068.03). 17β-Estradiol (E_2_) was purchased from Sigma-Aldrich (Cat# E2257). E_2_ was dissolved and stocked in ethanol at the concentration of 1 mM before addition into the culture medium. When being used for the treatment of cells, the final volume ratio of E_2_-ethanol solution to the medium was no more than 1:10,000 (V:V). The primary antibodies used in this study were purchased as follows: anti-PELP1, from Bethyl (Cat# IHC-00013); anti-ERα, from Santa Cruz (Cat# sc-543); anti-ERβ, from Abcam (Cat# ab288); anti-PCNA, from Boster (Cat# BM0104); anti-MMP-9, from Abcam (Cat# ab76003); anti-β-Actin, from Santa Cruz (Cat# sc-69879).

### Immunohistochemistry

Ten percent formalin-fixed and paraffin-embedded tissue blocks of the selected samples were retrieved from the archives for detecting PELP1, ERα, ERβ, PCNA, and MMP-9 protein expression. All samples were sliced to 4-μm thick serial section, deparaffinized with xylene, and rehydrated in alcohol gradients. Endogenous peroxidase was blocked with 3% hydrogen peroxide in methanol for 30 min. Antigen retrieval was performed by microwave (500 W) irradiation for 15 min. Primary antibodies against PELP1 (1/200), ERα (1/200), ERβ (1/100), PCNA (1/100), MMP-9 (1/100) were incubated overnight at 4°C. Real Envision Detection system (DAKO, Denmark, Cat# K5007) was used following the manufacturer’s instruction. Sections were visualized with DAB and counterstained with hematoxylin. Negative controls were performed by omitting the primary antibodies and substituting them with antibody dilution buffer (DAKO, Denmark, Cat# S2022).

The evaluation of immunoreactivity was performed independently by two of the authors (SC and ML). H-Score was used to assess PELP1 immunoreactivity. Briefly, PELP1 immunointensity was tentatively scored as 0, 1, 2, or 3, and the percentage of positive cells was determined for each score to yield a final score in the range of 0–300. The optimized cutoff points in the Habashy et al. [21] study were also employed in this study and the cut-off points were defined using the X-tile program. PELP1 immunoreactivity was tentatively classified into negative (H-score < 5), moderate (5 ≤ H-score < 170) and strong (170 ≤ H-score). When assessing ERα and ERβ immunoreactivity, tumor nuclear immunoreactivity of ≥1% was defined as positive. The evaluation of MMP-9 immunoreactivity followed the method reported previously [31]. When assessing PCNA immunoreactivity, the method of Grossi et al. was used [32].

### Transfection and Cell Models

Three siRNA sequences and nonspecific control (NC) siRNA targeting the PELP1 gene were designed and synthesized by Jima Pharmaceutical Technology (Shanghai, China). The cell transfections were performed using X-tremeGENE siRNA Transfection Reagent (Roche, Cat# 04-476-093-001) according to the manufacturers’ protocol.

### (3-(4, 5-Dimethylthiazol-2-yl)-2, 5-Diphenyltetrazolium Bromide (MTT) Assay

A549, H1975, and H-1299 cells were seeded in 96-well plates at a density of 1 × 10^4^ cells per well in complete phenol-red free DMEM medium with 5% charcoal stripped FBS under standard conditions (37°C and 5% CO_2_). After treatment with different treatment factors, a total of 20 μl MTT (5 mg/ml) was added to each well and incubated for additional 4 h, then the supernatant was aspirated and formazan crystals were dissolved in 0.5 M dimethylformamide and 20% sodium dodecylsulfate (SDS). Optical density (OD) was recorded at 490 nm using a microplate luminometer.

### Colony Formation Assay

A549 cells were seeded into six-centimeter plates in triplicates at a density of 600 cells/plate in complete phenol-red free DMEM medium and incubated for 24 h, then transfected with PELP1-siRNA-2 (siR-2) or NC for 24 h. The cells were then incubated with or without E_2_ for 2 weeks under standard conditions (37°C, 5% CO_2_ and 5% charcoal stripped FBS) and the culture medium with or without E_2_ was refreshed every 48 h. For counting the colonies, the cells were washed twice with phosphate-buffered saline (PBS), fixed with 5 ml methanol for 15 min and then stained with Giemsa for 30 min. Colonies containing more than 50 cells were counted under light microscopy with 100× magnification. The colony forming rate was calculated by dividing the number of positive colonies by the total number of cells seeded.

### Western Blot

Proteins were extracted with RIPA lysis buffer. Total 40 μg of protein for each sample was subjected to sodium dodecyl sulfate polyacrylamide gel electrophoresis (SDS-PAGE) and then transferred onto a Nitrocellulose blotting membrane. The membrane was blocked in 5% skimmed milk, then washed three times with Tris buffered saline with 0.05% Tween 20 (TBS-T), and probed with PELP1 (1/500), ERα (1/1000), ERβ (1/500), PCNA (1/200), MMP-9 (1/2000) or β-Actin (1/5000) primary antibodies overnight at 4°C. The samples were incubated with the appropriate secondary antibodies for 1 h. Quantification of the bands was analyzed with the NIH ImageJ program.

### Flow Cytometry

After treatment, the cells were re-suspended with 70% ethanol in a final concentration of 10^6^ cells/ml, and then incubated with propidium iodide (PI) at 4°C overnight. The cells in different cell cycle phases were counted on a FacScan (Becton Dickenson) with CellQuest software (Becton Dickenson) and analyzed using Cyflogic software (CyFlo Ltd.). The proportion of cells in each cell cycle phase was determined and the data were presented from three independent experiments.

### Wound Healing Assay

Cells were cultured in a monolayer reaching 70% confluence on six-centimeter plates. Monolayers were wounded by scratching with a 100-μl pipette tip and subsequently washed three times with PBS. During the treatment with E_2_ and/or siR-2, the images of the wound site were captured using a light microscopy (Ti, Nikon Instruments Inc., Japan) (magnification 200×) at the same location, respectively at 0 and 24 h. The cell migrated areas were then measured using NIH ImageJ program.

### Transwell Assay

Matrigel invasion assay was performed using 24-well transwell plates (Corning, Cat# 3422) with polycarbonate filters (pore size, 8 μm). Attenuated matrigel was made by diluting matrigel to 1 mg/ml with serum-free medium on ice (4°C). 100 μl of attenuated matrigel was added to the upper chamber of the transwell plates. Next, matrigel was rinsed using serum-free medium and 100 μl cell suspension (2 × 10^5^ cells/ml) was added to the upper chamber. For the lower chamber, 600 μl medium with 20% FBS was added. After 20–24 h of incubation in 37°C incubator, the transwell was washed with PBS twice and then the cells were fixed using 5% glutaraldehyde. Following fixation, the cells were rinsed twice in PBS and Giemsa staining was used to identify those invaded. The transwell was cultured at room temperature for 0.5 h and then washed in PBS twice. Finally, the invasion of cells in the transwell chambers was detected under light microscopy (90i, Nikon Instruments Inc., Japan) and invasive cells were calculated using the NIH ImageJ program.

### *In Silico* Data Analysis

Correlation between overall survival (OS) of LUAD patients and PELP1 expression (Affymetrix ID: 215354_s_at) was analyzed by the online cancer survival analysis tool Kaplan-Meier Plotter (http://kmplot.com). “Only JetSet best probe set” and “Auto select best cutoff” were selected in the analysis; Array quality control was set as “exclude biased array”. A total of 719 LUAD and 524 LUSC samples were included in the analysis. Kaplan-Meier analysis was performed to determine the correlation between the expression of PELP1 and OS of LUAD and LUSC patients.

### Statistical Analysis

Statistical analysis was performed with SPSS 17.0 statistical software (Chicago, United States). Association between PELP1 expression and clinicopathological variables in tissues was studied with Chi-square Test (Fishers Exact Test) or Spearman correlation test; ANOVA was used for analyzing results from *in vitro* assays (MTT, colony formation assay, western blot, flow cytometry, wound healing assay and transwell assay), and LSD method was chosen for Post Hoc Multiple Comparison. A *p* value < 0.05 was considered significant.

## Results

### Proline-, Glutamic Acid-, and Leucine-Rich Protein 1 in Lung Adenocarcinoma

**FIGURE 1:**
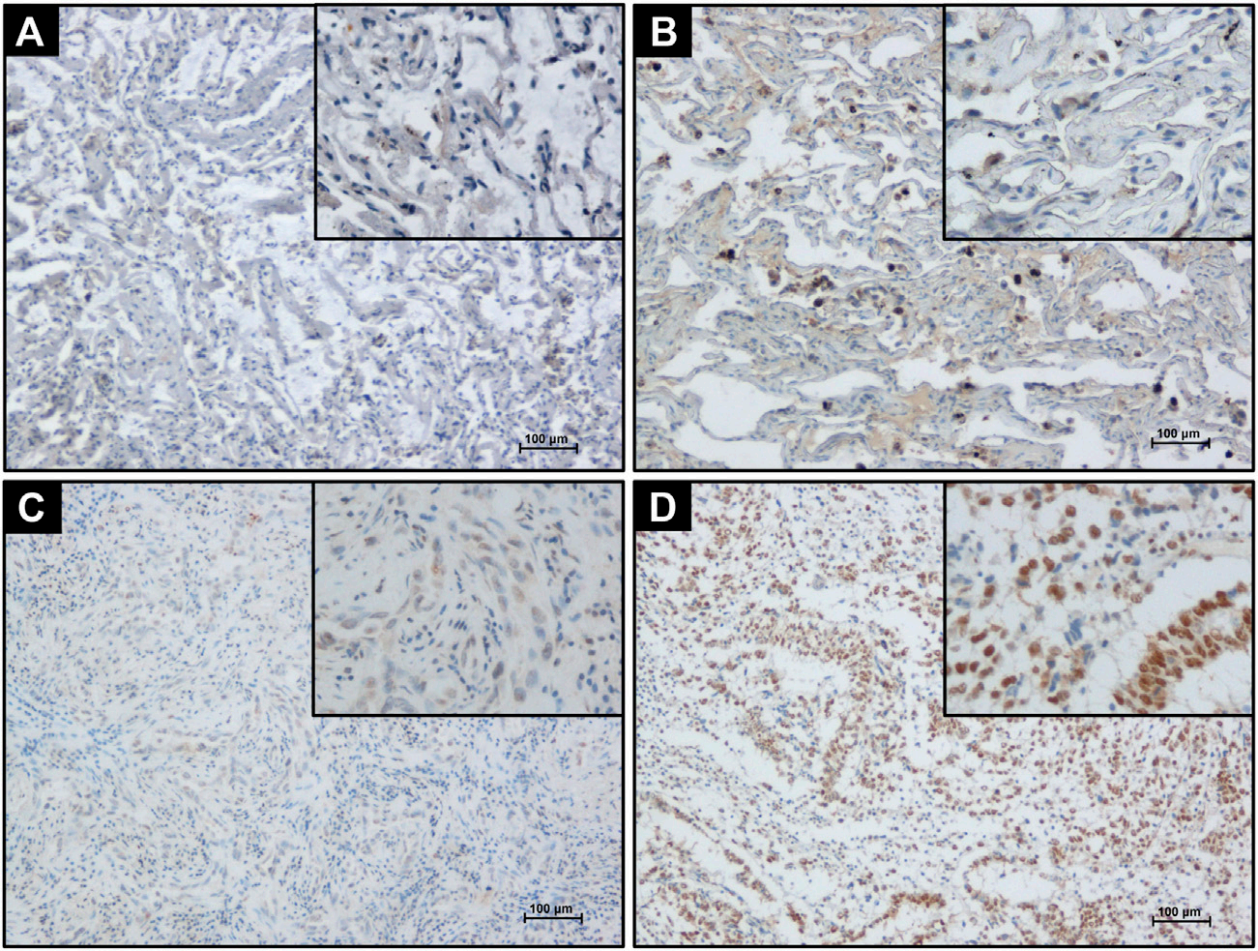
Detecting PELP1 expression in LUAD tissue by IHC. **(A)** Negative PELP1 immunostaining in NTL tissue. **(B)** Moderate nuclear PELP1 immunostaining in lung epithelium and lymphocytes in NTL tissue. **(C)** Weak nuclear PELP1 immunostaining in LUAD tissues. **(D)** Strong nuclear PELP1 immunostaining in LUAD tissues. Scale bar = 100 μm.

PELP1 immunoreactivity in 76 cases of LUAD and 17 cases of non-tumor lung (NTL) tissues were examined. In NTL tissues, PELP1 was immunohistochemically negative or moderate immunoreactivity in the nuclei of few lung epithelial cells and scattered lymphocytes. In LUAD tissues, PELP1 positive immunoreactivity was mainly located in the nuclei of tumor cells. In some cases, PELP1 moderate immunoreactivity could also be detected in some stromal fibroblast cells or lymphocytes. No cytoplasmic or extracellular immunostaining of PELP1 was detected in NTL or LUAD tissues (Figure 1).

Among the NTL cohort, 8/17 cases were immunohistochemically negative for PELP1, 9/17 moderately positive for PELP1 but none markedly positive. Among the LUAD cohort, 13/76 were negative for PELP1, 45/76 moderately positive, and 18/76 markedly positive. PELP1 status in LUAD was significantly higher than that in NTL (χ^2^ = 9.483, *p* = 0.006).

### Correlation of Proline-, Glutamic Acid-, and Leucine-Rich Protein 1 With ERα and ERβ as Well as Aggressive Phenotype of Lung Adenocarcinoma

**FIGURE 2:**
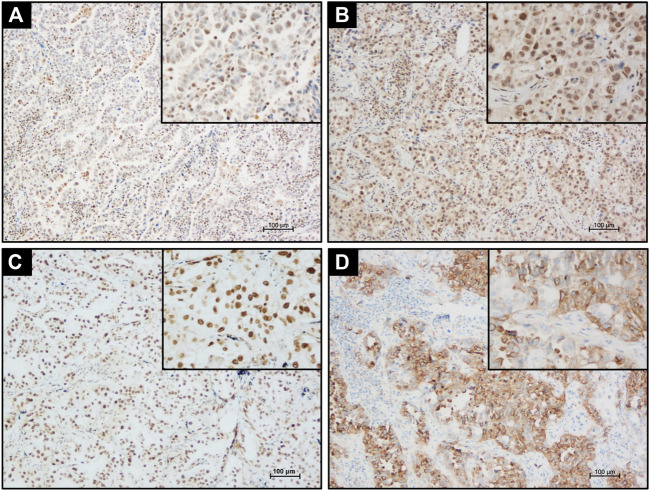
Detecting ERα, ERβ, PCNA, and MMP-9 expression in LUAD tissue by IHC. **(A)** Positive nuclear and cytoplasmic ERα immunostaining in LUAD tissues. **(B)** Positive nuclear ERβ immunostaining in LUAD tissues. **(C)** Strong nuclear PCNA immunostaining in LUAD tissues. **(D)** Strong cytoplasm MMP-9 immunostaining in LUAD tissue. Scale bar = 100 μm.

ERα, ERβ, PCNA, and MMP-9 were immunolocalized in the 76 cases of LUAD. Positive ERα immunoreactivity in LUAD cases was mainly located in the nuclei of tumor cells with its weak cytoplasmic immunoreactivity (Figure 2A) but ERβ immunoreactivity was only detected in the tumor nuclei without any cytoplasmic localization (Figure 2B). PCNA was exclusively immunolocalized in the nucleus (Figure 2C) and MMP-9 mainly distributed in the cytoplasm of LUAD cells (Figure 2D).

The status of PELP1 was correlated with clinicopathological variables of the patients including the status of ERα, ERβ, PCNA, and MMP-9 in LUAD. Significantly higher PELP1 was associated with tumors of higher grade with regard to the tumor differentiation, lymph node metastasis and clinical stage of the patients. In addition, PELP1 was significantly positively correlated with ERα, ERβ, and PCNA in LUAD patients but no significant association with other clinicopathological variables including patient’s gender, age, smoking history, tumor size as well as the expression of MMP-9 (Table 1).

**TABLE 1:** Correlated PELP1 expression to clinicopathological variables in LUAD.

Variables	*n*	Immunoreactivity of PELP1			χ^2^	*p*
		Negative (*n* = 13)	Moderate (*n* = 45)	Strong (*n* = 18)		
Gender						
Male	40	8	23	9	0.537	0.811
Female	36	5	22	9		
Age						
≤60	34	6	21	7	0.375	0.901
>60	42	7	24	11		
Smoking history						
Never	32	5	20	7	1.885	0.80
Ever	14	4	7	3		
Unknown	30	4	18	8		
Tumor size						
T1–T2	41	8	27	6	4.044	0.132
T3–T4	35	5	18	12		
Differentiation						
Well	23	6	15	2	11.657	0.017
Moderate	31	5	21	5		
Poor	22	2	9	11		
Lymph node metastasis						
Negative	30	7	21	2	7.159	0.016
Positive	46	6	24	16		
Stage						
I–II	35	6	26	3	8.747	0.012
III–IV	41	7	19	15		
ERα						
Negative	45	10	29	6	7.190	0.027
Positive	31	3	16	12		
ERβ						
Negative	37	9	24	4	7.631	0.022
Positive	39	4	21	14		
MMP-9						
Negative	31	8	21	2	11.123	0.068
Weak	19	3	10	6		
Moderate	15	1	9	5		
Strong	11	1	5	5		
PCNA						
Negative	21	7	12	2	11.060	0.022
Intermediate	21	4	14	3		
Intense	34	2	19	13		

### Overexpression of Proline-, Glutamic Acid-, and Leucine-Rich Protein 1 Promoted E_2_ Induced Proliferation and Migration of Lung Adenocarcinoma Cells

**FIGURE 3:**
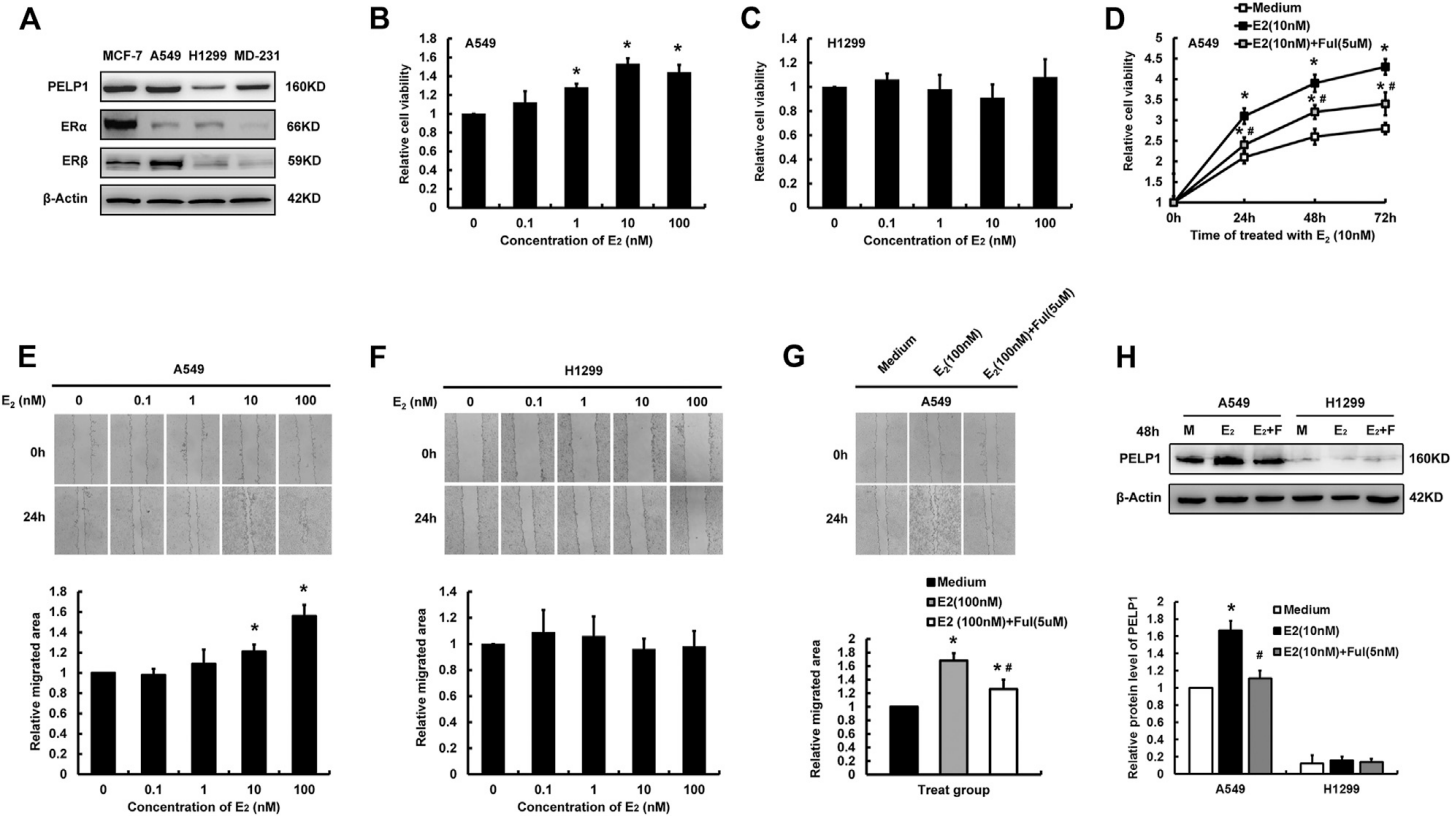
Influence of E_2_ on proliferation and migration of lung LUAD cells. **(A)** Detecting PELP1, ERα, and ERβ protein expression in different cells with western blot. **(B)** The influence of different concentration of E_2_ (treatment for 24 h) on the cell proliferation of A549. **(C)** The influence of different concentration of E_2_ (treatment for 24 h) on the cell proliferation of H-1299. **(D)** The influence of Ful on E_2_ induced proliferation of A549 cells. **(E)** The influence of different concentration of E_2_ on the cell migration of A549. **(F)** The influence of different concentration of E_2_ on the cell migration of H-1299. **(G)** The influence of Ful on E_2_ induced migration of A549 cells. **(H)** The influence of E_2_ with or without Ful on PELP1 protein expression of A549 cells, M (medium), F (Ful). Data were presented from three independent experiments; * compared with medium group, *p* < 0.05; # compared with E_2_ group, *p* < 0.05. Ful (fulvestrant).

In order to further study the functional roles of PELP1 in estrogen regulated proliferation and migration of LUAD cells, two LUAD cell lines, A549 and H-1299 were tentatively selected for the *in vitro* study in our present analysis. Western blot was first performed to examine PELP1, ERα, ERβ expression in MCF-7 (ERα, ERβ positive and PELP1 highly expressed) and MDA-MB-231 (ERα, ERβ negative and PELP1 highly expressed) breast carcinoma cells as control. A549 cells demonstrated high expression of PELP1 protein, nearly equivalent to that in MCF-7 and MDA-MB-231 cells but the protein level of PELP1 in H-1299 cells was significantly lower than that in MCF-7 and MDA-MB-231. A549 demonstrated weak ERα and robust ERβ expression, while in H-1299 cells, both ERα and ERβ were very low (Figure 3A).

MTT and wound healing assay were then performed to further explore the roles of estrogen on the proliferation and migration of two LUAD cells. Following the treatment with different concentrations of E_2_ for 24 h, A549 demonstrated an E_2_ concentration-dependent increment of proliferation and migration reaching their maximal effects, respectively at the concentration of 10 and 100 nM of E_2_ (Figures 3B,E). However, these pro-proliferation and pro-migration actions of E_2_ were not detected in H-1299 cells (Figures 3C,F). Treated A549 combining with 5uM Fulvestrant (Ful) significantly reduced the pro-proliferation and pro-migration activities of E_2_ on the cells (Figures 3D,G). The expression of PELP1 in A549 and H-1299 were examined by western blot after the cells were treated with E_2_ (10 nM) in combination with or without Ful (5 nM) for 48 h. In A549, the protein levels of PELP1were significantly increased after E_2_ treatment and inhibited by Ful treatment, but either E_2_ alone or in combination with Ful showed no influence on the PELP1 expression in H-1299 (Figure 3H). In addition, the pro-proliferation and pro-migration activities of E_2_ were also detected in ERs, PELP1 double positive H1975 LUAD cell line (Supplementary Figure S1). These results all indicated that both ERs and PELP1 were necessary for E_2_ to exert its pro-proliferation and pro-migration actions on LUAD cells.

### Knock Down of Proline-, Glutamic Acid-, and Leucine-Rich Protein 1 Gene Attenuated E_2_ Induced Cell Proliferation, Migration and Invasion of Lung Adenocarcinoma Cells

**FIGURE 4:**
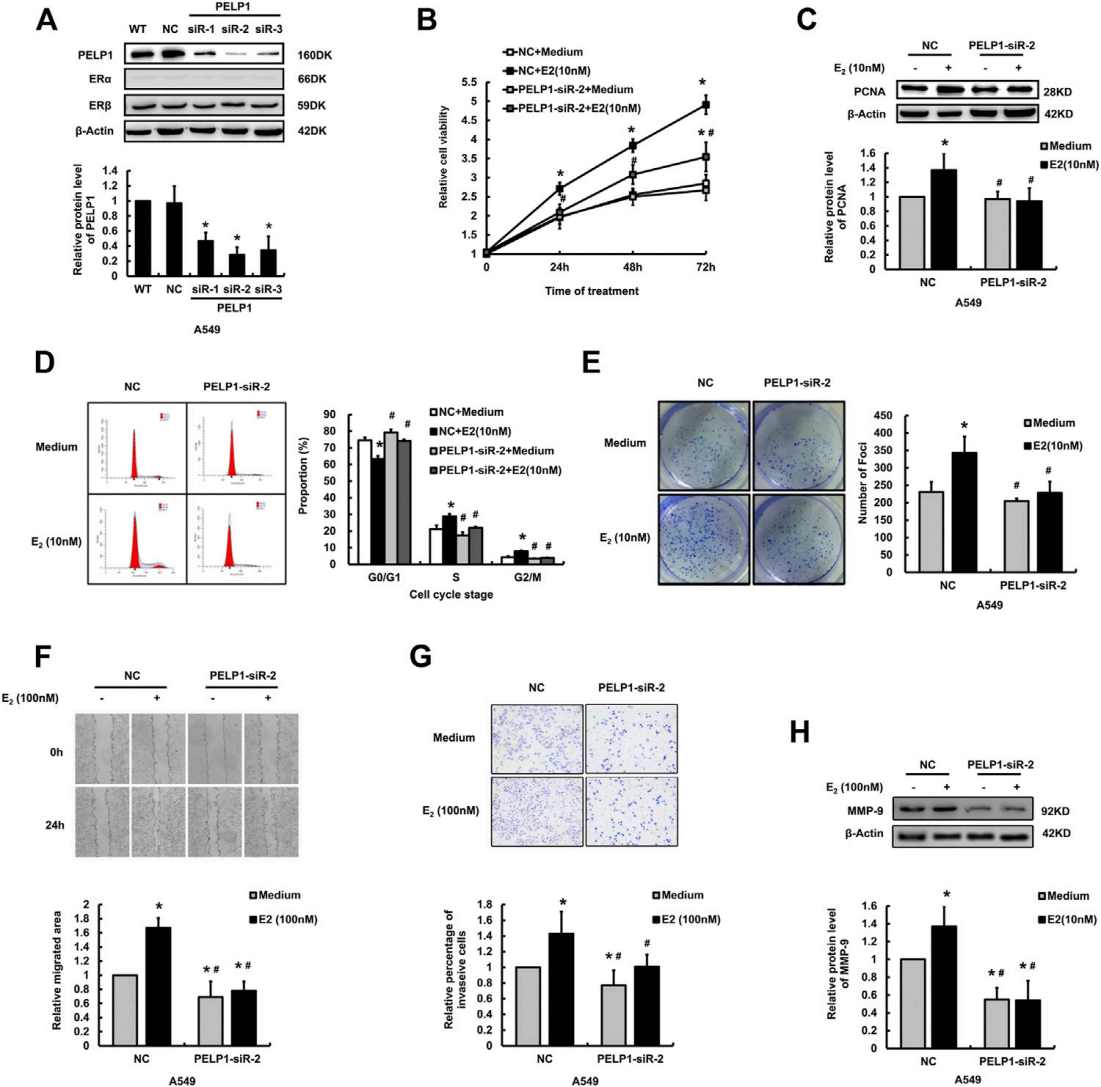
Influence of knocking down PELP1 on E_2_ induced proliferation and invasion of A549 cells. **(A)** Examining the protein levels of PELP1, ERα, and ERβ by western blot after the A549 cells were transfected with PELP1 siRNA. **(B)** Examining A549 cell proliferation activity by MTT test. **(C)** Examining PCNA protein levels by western blot. **(D)** Examining cell cycle by flow cytometry. **(E)** Examining cell growing ability formation with colony formation assay. **(F)** Examining cancer cell migration by wound healing assay. **(G)** Examining cancer cell invasion by transwell assay. **(H)** Examining MMP-9 protein levels by western blot. Data were presented from three independent experiments.* compared with control group, *p* < 0.05; # compared with E_2_ treated NC group, *p* < 0.05. WT (wild type), NC (non-specific RNA control).

In order to further identify the roles of PELP1 in the proliferation, migration, and invasion of A549 cells induced by E_2_, three siRNA sequences were designed targeting PELP1 in A549 cells. Results of western blot analysis indicated the three PELP1-siRNAs successful knocking down PELP1 and did by no means influence the expression of ERα or ERβ (Figure 4A). siR-2 demonstrated the highest knock-down efficiency and was therefore selected for subsequent analysis. MTT, western blot, and colony formation assay were all employed to assess the influence of knocking down PELP1on E_2_ induced proliferation of A549 cells. Under the stimulation of 10 nM E_2_, siR-2 transfected cells demonstrated significantly reduced cell viability compared with NC transfected cells in MTT assay (Figure 4B). Western blot assay also demonstrated that siR-2 prevented the increased PCNA protein level stimulated by E_2_ in A549 cells (Figure 4C). In addition, we also examined the cell cycle progress under the stimulation of E_2_ in NC or siR-2 transfected A549 cells. In the NC transfected A549 cells, E_2_ significantly increased the proportion of S and G2/M stage cells. However, in siR-2 transfected cells, E_2_ did not show significant influence on the cell cycle progress (Figure 4D). In addition, colony formation assay revealed knock-down of PELP1 using siR-2 significantly inhibited colony formation induced by E_2_ in A549 cells (Figure 4E). Both wound healing and transwell assays demonstrated that knock down of PELP1 significantly inhibited E_2_ (100 nM) induced migration and invasion of A549 cells (Figures 4F,G), and western blot also revealed that knock-down of PELP1 attenuated E_2_ induced MMP-9 expression (Figure 4H). Knocking down PELP1 in H1975 also attenuated E_2_ induced proliferation and migration as well as the expression of PCNA and MMP-9 of the cells (Supplementary Figure S2). Those results above all confirmed a pivotal role of PELP1 in E_2_ induced proliferation, migration, and invasion of LUAD cells.

### Overexpression of Proline-, Glutamic Acid-, and Leucine-Rich Protein 1 Correlated With a Worse Overall Survival of the Patients With Lung Adenocarcinoma

**FIGURE 5:**
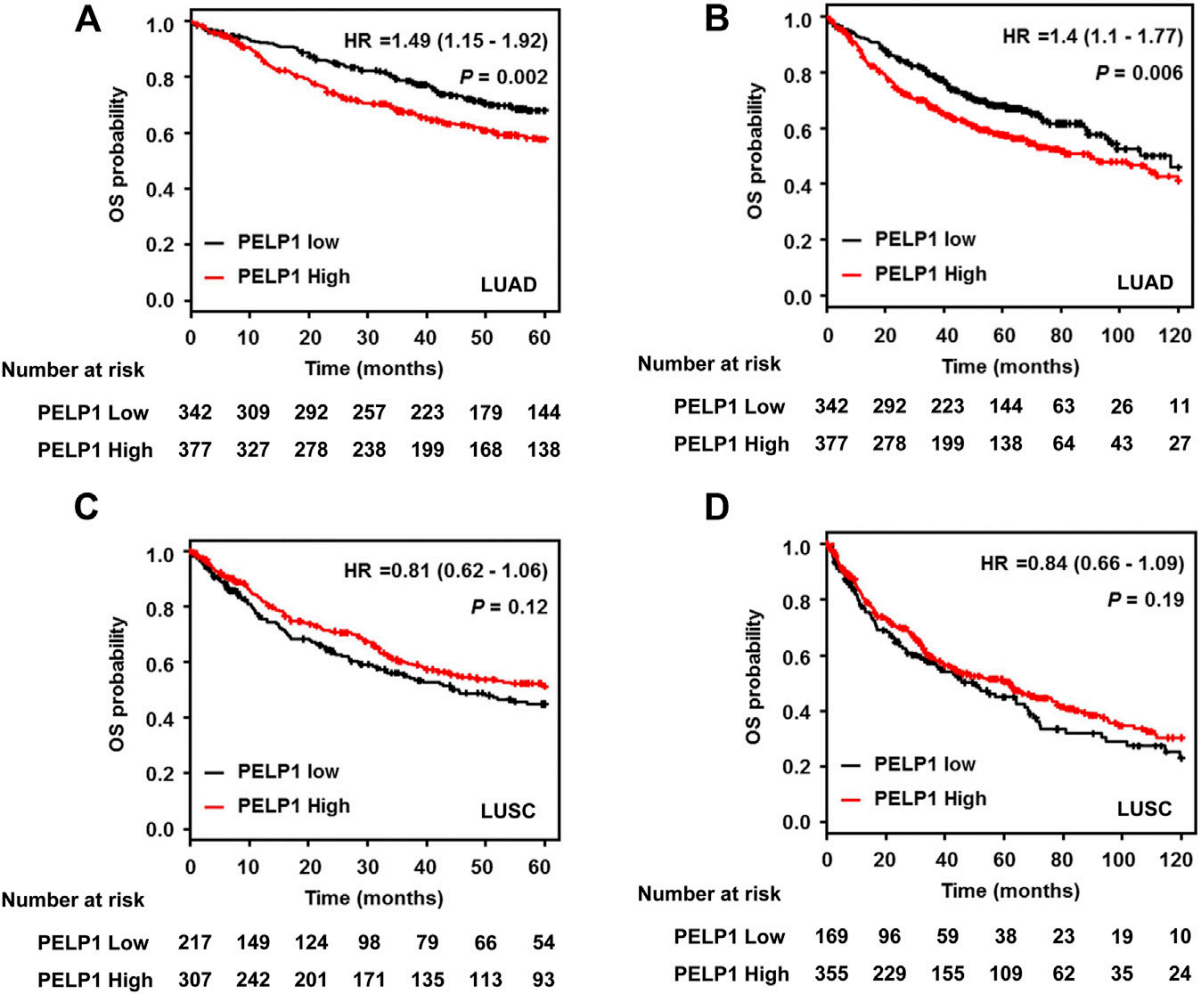
PELP1 expression and patients’ outcome in LUAD. Kaplan–Meier survival curve showed that LUAD patients with high PELP1 expression have significantly shorter OS than those with low PELP1 expression both in 5 years **(A)** and 10 years **(B)** follow-up; However, this difference wasn’t observed in LUSC group **(C, D)**.

In order to explore the potential prognostic value of PELP1 expression on LUAD patients, we performed survival analysis using the online survival analysis tool Kaplan-Meier Plotter. The Gene expression data and survival information utilized in the tool were based on the database of GEO, EGA, and TCGA. This *in silico* analysis demonstrated LUAD patients with high PELP1 expression had significantly shorter OS than those with low PELP1 expression (*p* = 0.002 for 60 months following-up, and *p* = 0.006 for 120 months following-up). However, this prognostic impact of PELP1 was not detected in the patients with LUSC (*p* = 0.12 for 60 months following up, and *p* = 0.19 for 120 months following up) (Figure 5).

## Discussion

The dysregulation of PELP1 expression in LUAD had been detected by RT-PCR and western blot, and primarily tested by IHC [29, 33] but the details of PELP1 in LUAD have remained virtually unknown. IHC provided important information as to assessing protein expression in tissues where parenchymal and stromal cells were intermixed and results could be easily applicable to clinical settings. In this study, we examined PELP1 in 76 cases of LUAD and 17 non-tumor lung (NTL) tissues using IHC and correlated the PELP1 IHC scores with clinicopathological parameters of the patients of LUAD. Slowikowski et al. reported elevated PELP1 expression in NSCLC by RT-PCR and western blot and results of our present study also demonstrated that PELP1 in LUAD was significantly higher than that in NTL tissues. In addition, the histological expression patterns of PELP1 in LUAD were identical to those in breast cancer cases, in which, PELP1 was reported to play a critical role in the initiation, development, and treatment resistance of the tumors. In this study, higher PELP1 expression was associated with tumors of higher grade with regard to tumor differentiation, lymph node metastasis, and clinical stage. These results all suggested that PELP1 could serve as a potential biomarker of LUAD aggressive phenotype.

PELP1 was reported to be correlated with the status of ERα or ERβ in different human malignancies. In this study, the association of PELP1 expression with ERs status in LUAD was also demonstrated. Being consistent with that in breast, ovarian and salivary duct adenocarcinoma [21, 24, 27], PELP1 in LUAD was significantly positively correlated with the status of ERα and ERβ. Correspondingly, *in vitro* study also demonstrated that the protein level of PELP1 was significantly increased in ERβ highly expressed A549 LUAD cells after being treated with 10 nM E_2_ for 48 h, and this increment could be blocked by the estrogen receptor down-regulator Fulvestrant (Ful). However, the PELP1 expression was not changed after E_2_ treatment in H-1299 negative for ERα or ERβ. Two estrogen-responsive element (ERE) half sites were reported in the promoter region of PELP1, and in the breast, endometrial as well as osteosarcoma model cancer cell lines, ERα or ERβ could be recruited to the promoter region of PELP1 and up-regulate the transcription of PELP1 [34]. These results at least in partial interpreted the intrinsic mechanisms of the positive correlation between PELP1 expression and ERs in cancer. Results of our present study also suggested that the regulatory roles of estrogen on the expression of PELP1 could be also present in the LUAD cells; the interaction between the estrogen signaling and PELP1 expression formed a positive feedback loop, therefore, promoted aggressive transformation of LUAD cells.

As a proto-oncogene, PELP1 promoted proliferation, migration, and invasion of multiple tumor cells. In breast carcinoma cells, under the stimulation of estrogen, PELP1 formed a complex with pRb, which subsequently facilitated CDKs and cyclin D1 induced phosphorylation of pRb, therefore, promoted the progress of cell cycling [18, 35]. In addition, PELP1could also enhance E_2_ induced ruffles and filopodium-like structure and up-regulated transcription of MMP-9 in breast cancer cells, resulting in the promotion of the metastasis of the tumor cells [36–38]. In lung cancer, Marquez-Garban et al. reported that PELP1 could be involved in the estrogen-induced proliferation of NCI-H23 NSCLC cells [28], and Ohshiro et al reported PELP1 played an important role in resveratrol-induced autophagy of lung carcinoma cells *via* interaction with LC3 in the autophagosomes [30]. In our present study, we validated the roles of PELP1 in pro-proliferation, migration, and invasion in LUAD with siRNA transfection and cytological assays. Results did reveal that knocking down PELP1with specific siRNA significantly suppressed E_2_ induced cell growth, cell cycle progress, colony formation, cell migration and invasion, as well as the expression of PCNA and MMP-9 in A549 LUAD cells. Results of our present study also confirmed the *in vitro* evidence and substantiated the proto-oncogenic role of PELP1 in LUAD.

PELP1 was reported as a prognostic biomarker of poorer outcomes in different human malignancies [21, 23–25]. In this study, the prognostic impact of PELP1 in LUAD was primarily tested with the online survival analysis tool Kaplan-Meier Plotter. An unfavorable prognostic significance of PELP1 in LUAD was revealed *in silico* that LUAD patients with high PELP1 expression demonstrated a significantly shorter OS than those with low PELP1 expression. It is noticeable that the 5-year survival rate (50–60%) of LUAD calculated by Kaplan-Meier Plotter was significantly higher than that we had known. We could not completely rule out the presence of bias in results from this *in silico* survival analysis. However, to our great regret, the further validation of this prognostic significance of PELP1 in LUAD could not be performed due to the insufficient follow-up information in our achieved tissue samples. Therefore, this is reasonably considered a major drawback of our present study and it awaits further clinicopathological studies for clarification.

H1975 cell line is characterized by EGFR mutations. Several studies reported treatment of the cells in combination with Ful or Tamoxifen significantly enhanced the cytotoxic effects of EGFR-tyrosine kinase inhibitors (EGFR-TKIs) including gefitinib and erlotinib [39–41]. In our present study, knock-down of PELP1 in H1975 significantly attenuated E_2_ induced cell proliferation and migration of the cells, suggesting that PELP1 could play an important role in the crosstalk between estrogen and EGF signaling. The expression of PELP1 is therefore considered to be correlated with the status of EGFR mutation in LUAD. However, to our regret, among 76 cases of LUAD cases obtained from three hospitals, data on EGFR were available only in 16 cases. Therefore, the cases in which EGFR status was examined was too small to draw any inert conclusions and we could not include those data in our present study. It awaits further investigations to clarify this aspect.

Taken together, in our present study in LUAD, we firstly demonstrated the expression patterns of PELP1, examined the correlation between the status of PELP1 and ERs, validated the potential functional roles of PELP1 in the proliferation, migration, and invasion of the tumor cells, and primarily explored the prognostic meaning of PELP1. The limited number of the cases available for our IHC study, the lack of exogenous PELP1 overexpression cell model and deficiency of further mechanistic study should be performed to provide more insights into the roles of PELP1 and contribute to the translational application of PELP1 in the potential personalized treatment of LUAD.

## Data Availability

The raw data supporting the conclusions of this article will be made available by the authors, without undue reservation.
